# Supplementary material for: Distribution of the Indicator of the Appropriate Admission of Patients with Circulatory System Diseases to County Hospitals in Rural China: A Cross-Sectional Study
Source: Int J Environ Res Public Health. 2019 May 9;16(9):1621. doi: 10.3390/ijerph16091621 (PMC6539859; doi:10.3390/ijerph16091621)
Supplement: Supplementary file 1 [file ijerph-16-01621-s001.pdf]

Supplementary Materials

Table S1. AEP criteria for county hospitalisation.

A.	Requirement for services
1.	Need surgery or follow-up treatment within 24 hours: (1) local anesthesia or general anesthesia; and/or (2) instruments or other facilities that are only available for hospitalized patients (angiography, visceral biopsy) (Abbreviation: need surgery/follow-up treatment within 24 hours)
2.	Treatment with varying dosage or drug on a regular basis under direct medical supervision (Abbreviation: Varying dosage/drug under supervision)
3.	Calculation of intake and output volume (Abbreviation: Calculate intake/output volume)
4.	Operation to be conducted on the following day in the operating room, detailed pre-operative consultation or evaluation on the day of admission (Abbreviation: Operation on the following day)
5.	Main surgical incision and drainage nursing (Abbreviation: Surgical incision & drainage nursing)
6.	Quarantined patients
7.	Bedside electrocardiogram (ECG) monitoring or testing vital signs at least every 2 hours (Abbreviation: ECG per 2 hours)
8.	Stopping (at least once every 8 hours) or continuing oxygen inhalation (Abbreviation: Stopping/continuing oxygen inhalation)
9.	Referral of post-operative recovery
**B.**	**Diseases severity**
1.	Continuous fever>38.0°C for more than 5 days (Abbreviation: fever>38.0°C & > 5 days)
2.	Acute confusion (coma or adiaphoria) (Abbreviation: Acute confusion)
3.	Severe anomaly in electrolyte or blood and vigor, showing the following situations: (1) Na<123 mEq/L or>156 mEq/L; (2) K<2.5 mEqt/L or>6.0 mEq/L; (3) HCO3 <20 mEq/L or>36 mEq/L; and (4) arterial blood pH<7.30 or>7.45 (Abbreviation: Severe in electrolyte / blood and vigor)
4.	Loss of sight or hearing for 48 hours (Abbreviation: sight or hearing losing for 48 hours)
5.	Loss of activity in any part of the body for 48 hours (Abbreviation: Partial Physical inactivity within 48 hours)
6.	Excretion disorder or absence of intestinal peristalsis in the past 24 hours (Abbreviation: Abnormal excretion in the past 24 hours)
7.	Active bleeding
8.	Needing blood transfusion because of bleeding (Abbreviation: Needing blood transfusion)
9.	Mental disorders caused by non-alcohol dependence (Abbreviation: Non-alcohol mental disorders)
10.	Viscera removal or surgical wound dehiscence (Abbreviation: Dehiscence of surgical wound)
11.	Pulse less than 50 times or greater than 140 times per minute (Abbreviation: Abnormal pulse)
12.	Abnormal blood pressure: systolic blood pressure<90 mmHg or>200 mmHg and/or diastolic blood pressure<60 mmHg or>120 mmHg (Abbreviation: Abnormal blood pressure)

13.	Ventricular fibrillation or acute myocardial ischemia shown by electrocardiogram (ECG) report or course log (Abbreviation: Ventricular fibrillation/acute myocardial ischemia)
14.	Acute blood disorder, severe medium-sized leukopenia, thrombocytopenia, leukocytosis, erythrocytosis, thrombocytosis or hemolysis-resulted symptoms (Abbreviation: Abnormal blood condition)
15.	Progressive acute neurological disorders
16.	Soft tissue injuries affecting basic self-care (Abbreviation: Soft tissue injuries)
17.	Acute myocardial infarction or cerebrovascular accident (stroke) (Abbreviation: Acute myocardial infarction/stroke)
18.	Spinal cord lesions
19.	Lung infection above 40% or leafy lesions according to X-ray examination (Abbreviation: Lung infection > 40% or leafy lesions)
20.	Hyperemesis or acute pain at acute attack by chronic diseases (Abbreviation: Acute episode symptoms of chronic diseases)
21.	Burns occurred in specific areas (Abbreviation: Burns)
